# Impact of Virtual Touch Quantification in Acoustic Radiation Force Impulse for Skeletal Muscle Mass Loss in Chronic Liver Diseases

**DOI:** 10.3390/nu9060620

**Published:** 2017-06-16

**Authors:** Hiroki Nishikawa, Takashi Nishimura, Hirayuki Enomoto, Yoshinori Iwata, Akio Ishii, Yuho Miyamoto, Noriko Ishii, Yukihisa Yuri, Ryo Takata, Kunihiro Hasegawa, Chikage Nakano, Kazunori Yoh, Nobuhiro Aizawa, Yoshiyuki Sakai, Naoto Ikeda, Tomoyuki Takashima, Shuhei Nishiguchi, Hiroko Iijima

**Affiliations:** Division of Hepatobiliary and Pancreatic disease, Department of Internal Medicine, Hyogo College of Medicine, Hyogo 6638501, Japan; nishikawa_6392@yahoo.co.jp (H.N.); tk-nishimura@hyo-med.ac.jp (T.N.); yo-iwata@hyo-med.ac.jp (Y.I.); akio0010@yahoo.co.jp (A.I.); yuho.0818.1989@gmail.com (Y.M.); ishinori1985@yahoo.co.jp (N.I.); gyma27ijo04td@gmail.com (Y.Y.); chano_chano_rt@yahoo.co.jp (R.T.); hiro.red1230@gmail.com (K.H.); chikage@hyo-med.ac.jp (C.N.); mm2wintwin@ybb.ne.jp (K.Y.); nobu23hiro@yahoo.co.jp (N.A.); sakai429@hyo-med.ac.jp (Y.S.); nikeneko@hyo-med.ac.jp (N.I.); tomo0204@yahoo.co.jp (T.T.); nishiguc@hyo-med.ac.jp (S.N.); hiroko-i@hyo-med.ac.jp (H.I.)

**Keywords:** virtual touch quantification, skeletal muscle mass, bio-electronic impedance analysis, liver fibrosis marker, predictive ability

## Abstract

Background and aims: We sought to clarify the relationship between virtual touch quantification (VTQ) in acoustic radiation force impulse and skeletal muscle mass as assessed by bio-electronic impedance analysis in patients with chronic liver diseases (CLDs, *n* = 468, 222 males and 246 females, median age = 62 years). Patients and methods: Decreased skeletal muscle index (D-SMI) was defined as skeletal muscle index (SMI) <7.0 kg/m^2^ for males and as SMI <5.7 kg/m^2^ for females, according to the recommendations in current Japanese guidelines. We examined the correlation between SMI and VTQ levels and investigated factors linked to D-SMI in the univariate and multivariate analyses. The area under the receiver operating curve (AUROC) for the presence of D-SMI was also calculated. Results: In patients with D-SMI, the median VTQ level was 1.64 meters/second (m/s) (range, 0.93–4.32 m/s), while in patients without D-SMI, the median VTQ level was 1.11 m/s (range, 0.67–4.09 m/s) (*p* < 0.0001). In the multivariate analysis, higher VTQ was found to be an independent predictor linked to the presence of D-SMI (*p* < 0.0001). In receiver operating characteristic analysis, body mass index had the highest AUROC (0.805), followed by age (0.721) and VTQ (0.706). Conclusion: VTQ levels can be useful for predicting D-SMI in patients with CLDs.

## 1. Introduction

The severity of liver fibrosis in patients with chronic liver diseases (CLDs) is the major determinant of long-term clinical outcomes, driving both the development of liver-related complications and mortality [1,2,3,4]. Thus, evaluating the degree of liver fibrosis correctly plays a pivotal role for the control of disease progression and for creating treatment strategies and evaluating the prognosis for CLD patients [5,6]. High diagnostic accuracy, easy accessibility, and the possibility for follow-up examinations can lead to the implementation of non-invasive diagnostic methods into daily clinical practice.

Acoustic radiation force impulse (ARFI), which is a technology designed to measure shear wave-front at multiple sites to estimate tissue stiffness, is a novel ultrasound-based radiological technique for non-invasively assessing the degree of tissue stiffness [7,8,9,10]. This modality can evaluate the degree of liver fibrosis easily and accurately in the clinical settings [10,11]. ARFI elastography has two modes which involve qualitative response and quantitative response for virtual touch quantification (VTQ), and it measures transverse shear wave velocity values in meters/second (m/s). The shear wave velocity reflects tissue stiffness through a simple method: the stiffer the tissue, the higher shear wave velocity [7,8,9,10,11]. A recent meta-analysis demonstrated that ARFI elastography was a good method for evaluating the degree of liver fibrosis and had similar predictive value to transient elastography for significant fibrosis and liver cirrhosis (LC) [12]. Another recent study reported that spleen stiffness measured by ARFI imaging can predict clinical outcomes for LC patients with high accuracy [13]. On the other hand, several serum liver fibrosis markers for predicting the degree of liver fibrosis have been proposed and validated as well as radiological modalities for assessing liver fibrosis [12,14,15,16]. Of these, FIB-4 index and aspartate aminotransferase (AST) to platelet ratio index (APRI) have been the most widely utilized liver fibrosis markers in CLD patients [16,17,18,19,20,21].

On the other hand, skeletal muscle is regarded as having a role in maintaining energy metabolism and nutritional status, and the depletion of skeletal muscle mass may be a considerable impairment circumstance [22,23,24,25]. Skeletal muscle mass loss can be affected by aging, while LC is occasionally associated with this muscular disorder [22,23]. In our previous investigation, the percentage of skeletal muscle mass loss as determined by bio-electronic impedance analysis (BIA) in LC subjects was significantly higher than that in subjects with chronic hepatitis without LC [26]. Other studies have claimed the significant role of muscle mass assessment in LC patients as a helpful marker for reflecting malnutrition and liver function and for predicting clinical outcomes [26,27,28,29,30]. Recently, sarcopenic obesity, which is the coexistence of sarcopenia as defined by low muscle mass and muscle strength and obesity, has been treated with much caution due to its close linkage to clinical outcomes in CLD patients [31,32].

Considering these reports, the speculation that liver fibrosis marker level is associated with the development of skeletal muscle loss in patients with CLDs may be true. Several reports showing that the proportion of decreased skeletal muscle mass (D-SMI) demonstrated a linear increment with liver fibrosis progression in non-alcoholic fatty liver disease (NAFLD) or non-alcoholic steatohepatitis (NASH) patients support our hypothesis [33,34,35]. However, an extensive literature search has not shown the relationship between skeletal muscle mass and the liver fibrosis markers in CLD patients, and these clinical questions should be fully addressed. In this study, we sought to clarify the relationship between radiological or serum liver fibrosis markers (VTQ, FIB-4 index, APRI, and platelet count) and skeletal muscle mass, as well as to investigate factors linked to the decrease in skeletal muscle mass in CLD patients.

## 2. Patients and Methods

### 2.1. Patients

In this retrospective study, we analyzed 549 patients with CLD who were admitted to the Division of Hepatobiliary and Pancreatic disease, Department of Internal Medicine, Hyogo College of Medicine, Hyogo, Japan between October 2008 and May 2014, and were assessed using BIA to diagnose decreased skeletal muscle mass. All CLD patients who agreed to nutritional evaluation were included. Next, patients without data for VTQ (*n* = 48), those with advanced malignancies which potentially affect the development of muscle mass loss (*n* = 16), and those with severe ascites (*n* = 17) were excluded. Patients with severe ascites were excluded because body weight and body mass index (BMI) may be overestimated in these patients, and skeletal muscle index (SMI) may also be overestimated by BIA [26]. Several edematous patients with advanced cirrhosis (Child-Pugh B or C), hypoalbuminemia, and severe ascites were thus excluded from the current analysis. A total of 468 CLD patients were therefore analyzed. In this study, 447 patients underwent liver biopsy. For patients without liver biopsy data (*n* = 21), LC was determined by radiological findings such as deformity of the liver surface and presence of varices. We assessed skeletal muscle mass by employing SMI using BIA at baseline. SMI indicates appendicular skeletal muscle mass divided by height squared (cm^2^/m^2^). D-SMI was defined as SMI <7.0 kg/m^2^ for males and as SMI <5.7 kg/m^2^ for females, according to the recommendations in current Japanese guidelines [26]. We examined the correlation between SMI and liver fibrosis markers (VTQ, FIB-4 index, APRI, and platelet count) and investigated factors linked to D-SMI in the univariate and multivariate analyses.

APRI score was calculated as reported elsewhere: AST level/upper limit of normal level for AST/platelet count (×10^9^/L) × 100 [17,18,19,20]. The FIB-4 index was calculated as reported elsewhere: age (years) × AST (IU/L) /platelet count (×10^9^/L) ×√alanine aminotransferase (ALT) (IU/L) [19,20,21]. Our protocols for liver biopsy were detailed in our previous study, and the degrees of liver fibrosis and inflammation were determined as reported elsewhere [36]. The ethics committee meeting of Hyogo college of medicine acknowledged this study protocol (approval number: 2117, approval date: 1 March 2016). And our study protocol conformed to all of the regulations of the Declaration of Helsinki.

### 2.2. Measurement for VTQ

We have routinely used a Siemens ACUSON S2000/3000 (Mochida Siemens Medical Systems, Tokyo, Japan) for VTQ measurement. The method for VTQ measurement by ARFI was conducted as reported elsewhere [10]. Briefly, using intercostal approach by one experienced sonologist, the examination for VTQ measurement was performed on the right lobe of the liver with a measurement depth of 2–3 cm below the liver surface. When successful acquisitions for VTQ data six times at different sites in the liver were obtained on each subject, the median value of these was calculated and data were presented in m/s [10].

### 2.3. Statistical Analysis

For quantitative parameters, the statistical analysis between groups was performed using Student’s *t* test, Mann-Whitney U-test, Kruskal-Wallis test, Fisher’s exact test, or Spearman’s rank correlation coefficient *r_s_* as appropriate. Parameters with *p* value < 0.05 in the univariate analysis were entered into the multivariate analysis utilizing the logistic regression analysis. In the multivariate analyses, significant variables in the univariate analyses were changed to dichotomous covariates using each median value. Receiver operating characteristic curve (ROC) analysis for the presence of D-SMI was performed for calculating the area under the ROC (AUROC) in baseline quantitative variables. *p* values of less than 0.05 were considered to suggest significance. Data are presented as median values (range) unless otherwise mentioned. Statistical analysis was performed with the JMP 11 (SAS Institute Inc., Cary, NC, USA).

## 3. Results

### 3.1. Baseline Characteristics

**Figure 1:**
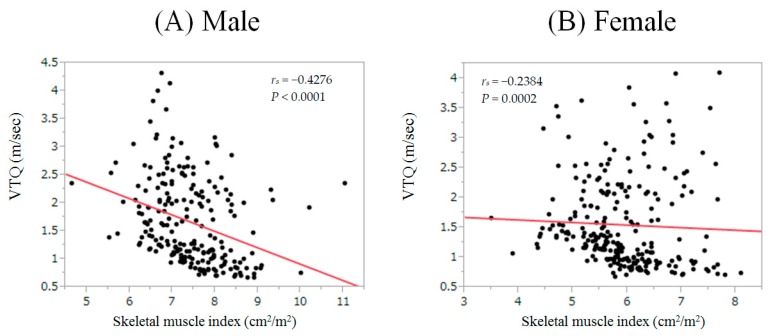
(**A**) Correlation between virtual touch quantification (VTQ) and skeletal muscle index (SMI) in males. Significant inverse correlation between VTQ and SMI was found (*r_s_* = −0.4276, *p* < 0.0001); (**B**) Correlation between VTQ and SMI in females. Significant inverse correlation between VTQ and SMI was found (*r_s_* = −0.2384, *p* = 0.0002).

The baseline characteristics of the analyzed subjects (*n* = 468) are shown in Table 1. There are 222 males and 246 females with the median (range) age of 62 (18–90) years. In terms of liver fibrosis stages, F4 was observed in 178 patients, F3 in 73, F2 in 73, F1 in 114, F0 in 9, and not tested in 21. Patients predominantly presented with hepatitis C virus (HCV) infection (57.1%, 267/468). The median (range) SMI for males and females were 7.35 (4.66–11.05) cm^2^/m^2^ and 5.86 (3.50–8.10) cm^2^/m^2^, respectively. In this analysis, there were 76 male patients with D-SMI (33.3%) and 99 female patients with D-SMI (40.2%) (*p* = 0.1823). VTQ ranged from 0.67 m/s to 4.32 m/s (median, 1.34 m/s). FIB-4 index ranged from 0.12 to 2.61 (median, 1.03). APRI ranged from 0.09 to 7.58 (median, 0.83). In males, a significant inverse correlation between VTQ and SMI was found (*r_s_* = −0.4276, *p* < 0.0001). Similarly, in females, a significant inverse correlation between VTQ and SMI was found (*r_s_* = −0.2384, *p* = 0.0002) (Figure 1A,B).

### 3.2. VTQ Level according to The Degree of Liver Fibrosis

**Figure 2:**
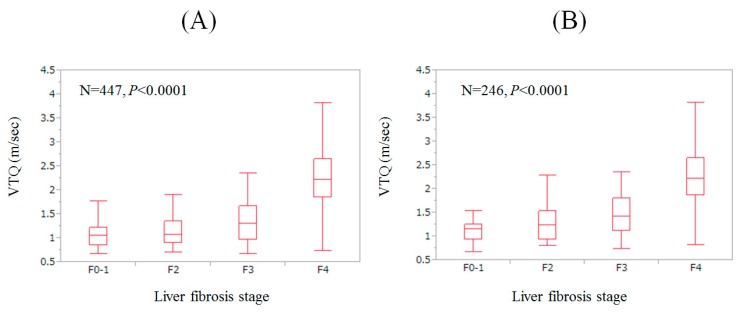
(**A**) VTQ level according to liver fibrosis stage for the entire cohort (excluding 21 patients with missing data for liver histology, *n* = 447). The stepwise increase of VTQ level was found according to the severity of liver fibrosis (overall significance, *p* < 0.0001); (**B**) VTQ level according to liver fibrosis stage for patients with hepatitis C virus (HCV) (excluding patients with missing data for liver histology, *n* = 246). The stepwise increase of VTQ level was found according to the severity of liver fibrosis (overall significance, *p* < 0.0001).

For the entire cohort (excluding 21 patients with missing data for liver histology, *n* = 447), the stepwise increase of VTQ level was found according to the severity of liver fibrosis (overall significance, *p* < 0.0001). (Figure 2A) Similarly, for patients with HCV (excluding patients with missing data for liver histology, *n* = 246), the stepwise increase of VTQ level was also found according to the severity of liver fibrosis (overall significance, *p* < 0.0001) (Figure 2B).

### 3.3. VTQ Level in Patients with and without D-SMI

**Figure 3:**
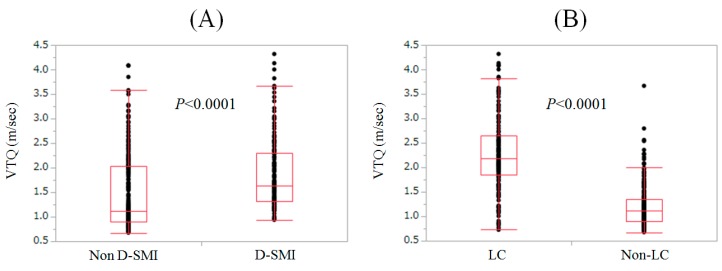
(**A**) VTQ level in patients with and without decreased skeletal muscle mass (D-SMI). In patients with D-SMI, the median VTQ level was 1.64 m/s (range, 0.93–4.32 m/s), while in patients without D-SMI, the median VTQ level was 1.11 m/s (range, 0.67–4.09 m/s) (*p* < 0.0001); (**B**) VTQ level according to liver cirrhosis (LC) status. In patients with LC, the median VTQ level was 2.18 m/s (range, 0.73–4.32 m/s), while in patients without LC, the median VTQ level was 1.11 m/s (range, 0.67–3.67 m/s) (*p* < 0.0001).

In patients with D-SMI, the median VTQ level was 1.64 m/s (range, 0.93–4.32 m/s), while in patients without D-SMI, the median VTQ level was 1.11 m/s (range, 0.67–4.09 m/s) (*p* < 0.0001) (Figure 3A).

### 3.4. VTQ Level according to LC Status

In patients with LC, the median VTQ level was 2.18 m/s (range, 0.73–4.32 m/s), while in patients without LC, the median VTQ level was 1.11 m/s (range, 0.67–3.67 m/s) (*p* < 0.0001) (Figure 3B).

### 3.5. ROC Analysis for the Presence of D-SMI

Results for ROC analyses (AUROC, optimal cutoff point, sensitivity, and specificity) for the presence of D-SMI are presented in Table 2. BMI had the highest AUROC (0.805), followed by age (AUROC = 0.721), VTQ (AUROC = 0.706), and serum albumin (AUROC = 0.698).

### 3.6. Relationship between VTQ and Baseline Variables

Age (*r_s_* = 0.4418, *p* < 0.0001), AST (*r_s_* = 0.3315, *p* < 0.0001), total bilirubin (*r_s_* = 0.1994, *p* < 0.0001), serum ammonia (*r_s_* = 0.3191, *P* < 0.0001), FIB-4 index (*r_s_* = 0.2122, *p* = 0.0001), and APRI (*r_s_* = 0.6011, *p* < 0.0001) had significantly positive correlations with VTQ level. SMI in males (*r_s_* = −0.4276, *p* < 0.0001), SMI in females (*r_s_* = −0.2384, *p* = 0.0002), serum albumin (*r_s_* = −0.5538, *p* < 0.0001), prothrombin time (*r_s_* = −0.5504, *p* < 0.0001), platelet count (*r_s_* = −0.6152, *p* < 0.0001), total cholesterol (*r_s_* = −0.3941, *p* < 0.0001), triglyceride (*r_s_* = −0.2075, *p* < 0.0001), and branched-chain amino acid to tyrosine ratio (BTR) (*r_s_* = −0.6061, *p* < 0.0001) had significant inverse correlations with VTQ level (Table 3).

### 3.7. Univariate and Multivariate Analyses of Factors Contributing to The Presence of D-SMI

Significant variables linked to the presence of D-SMI in the univariate analyses are: age (*p* < 0.0001); BMI (*p* < 0.0001); serum albumin (*p* < 0.0001); platelet count (*p* = 0.0044); BTR (*p* = 0.0219); VTQ (*p* < 0.0001); FIB-4 index (*p* < 0.0001); and APRI (*p* = 0.0204) (Table 4). The odds ratios (ORs) and 95% confidence intervals calculated by using multivariate analysis for the eight significant parameters (*p* < 0.05) in the univariate analysis are presented in Table 5. BMI (*p* < 0.0001), age (*p* < 0.0001), serum albumin (*p* < 0.0001), BTR (*p* = 0.0031), VTQ (*p* < 0.0001), and FIB-4 index (*p* = 0.0425) were found to be independent predictors associated with the presence of D-SMI (Table 5).

## 4. Discussion and Conclusions

Radiological and serum liver fibrosis markers are useful markers and have been preferably used in the clinical settings for their non-invasiveness and easy availability [7,8,9,10,11,13]. Indeed, the stepwise increase of VTQ level according to the severity of liver fibrosis was found in our data. However, there have been no available data for the relationship between skeletal muscle mass and VTQ level in CLD patients. Because skeletal muscle mass in CLDs has been drawing much caution due to its prognostic significance, addressing this problem may be clinically essential [23,26,28,29,30]. We therefore conducted the current analysis. To the best of our knowledge, this is the first report linking skeletal muscle loss with liver damage in CLDs in a Japanese population using data for VTQ.

In our results, a significant inverse correlation between SMI and VTQ level in both males and females, and a significant difference of VTQ level between patients with and without D-SMI were found. Additionally, AUROC of VTQ for D-SMI was 0.706, and higher VTQ levels were revealed to be significantly associated with the development of D-SMI in the multivariate analysis (*p* < 0.0001). These results denote that VTQ level can be helpful for not only assessing the degree of liver fibrosis, but also for predicting D-SMI. A recent Korean study (*n* = 309) demonstrated that significant stratification was found in terms of the proportion of sarcopenia as evaluated by the appendicular skeletal muscle mass divided by body weight among subjects with normal livers, those with NAFLD, and those with NASH, which were in line with our current data [35].

Although LC-related muscle mass loss is defined as secondary sarcopenia as well as chronic inflammatory diseases and advanced malignancies, aging can cause D-SMI in CLDs at least in part [24,26]. The significant positive correlation between age and VTQ level (*r_s_* = 0.4418, *p* < 0.0001) may be associated with our current results. The fact that FIB-4 index includes age may also well explain the statistical significance of FIB-4 index for predicting D-SMI in the multivariate analysis. As a greater number of Japanese CLD patients have been aging in recent years, this is a critical problem.

BTR had the second strongest correlation with VTQ level (*r_s_* = −0.6061) and was revealed to be an independent predictor for the development of D-SMI (*p* = 0.0031). The liver is a central organ for the metabolism of three major classes of molecules: fats, proteins, and carbohydrates [37,38]. Deterioration in liver functional reserve can be accompanied by numerous nutritional disorders, and BTR reflects the nutritional state [39,40]. Poor nutritional status can cause muscle mass depletion. Branched-chain amino acid granules are promising for ameliorating muscle mass depletion [28]. In a sense, VTQ level may be helpful for assessing nutritional state in CLD patients. It is also of note that VTQ level had significant positive correlation with serum ammonia level (*r_s_* = 0.3191, *p* < 0.0001). Elevated serum ammonia levels lead to the suppression of protein synthesis in the muscle [6,23,26].

In our results, lower BMI had strong impact on D-SMI, while in the Korean national study with large NAFLD cohort (*n* = 2761), the proportion of sarcopenia using dual-energy X-ray absorptiometry was prominent in obese patients [34]. The average BMIs in our study and their study were 22.8 kg/m^2^ and 25.8 kg/m^2^, respectively. In our cohort, 109 patients (23.3%) had BMI >25 kg/m^2^ and only five patients (1.1%) had BMI >35 kg/m^2^. The distribution of BMI in baseline characteristics between these studies may lead to such different results. The incidence of sarcopenic obesity has been increasing and the relation between muscle mass and BMI should be further examined in future studies [31,32].

We acknowledge several weak points to our study. First, this is a retrospective single center Japanese study. Thus, whether our data can be applied to other ethnic backgrounds remains unclear. Second, in this cross-sectional study, we are unable to interpret any causal relationship; in other words, an in-depth mechanism for the independent association between D-SMI and liver fibrosis was not clarified in our study. Third, liver biopsy has a significant limitation for sampling errors in evaluating the severity of liver fibrosis. Finally, patients with severe ascites who are expected to have D-SMI with higher rates were excluded from our analysis, potentially leading to bias. Caution should therefore be exercised when interpreting our results. However, our current results presented that VTQ can be useful for predicting D-SMI in CLD patients. In our previous investigation, we reported that a predictive model involving VTQ level is promising for assessing the risk of liver carcinogenesis [10]. Thus, we anticipate that VTQ involves various clinical significances for CLD patients.

In conclusion, VTQ level can provide useful insights for predicting D-SMI in patients with CLDs.

## Figures and Tables

**Table 1:** Baseline characteristics (*n* = 468).

Variables	Number or Median (Range)
Age (years)	62 (18–90)
Gender, male/female	222/246
SMI (cm^2^/m^2^), male	7.35 (4.66–11.05)
SMI (cm^2^/m^2^), female	5.86 (3.50–8.10)
BMI (kg/m^2^)	22.26 (13.05–41.94)
Cause of liver disease, B/C/B and C/alcoholic/others	47/267/4/17/133
AST (IU/L)	36 (12–182)
ALT (IU/L)	35 (7–268)
Serum albumin (g/dL)	3.9 (2.6–5.1)
Total bilirubin (mg/dL)	0.9 (0.2–5.1)
Prothrombin time (%)	89.8 (43.3–133.6)
Platelet count (×10^4^/mm^3^)	14.9 (2.1–50.4)
Total cholesterol (mg/dL)	167 (82–448)
Triglyceride (mg/dL)	91 (27–572)
Fasting blood glucose (mg/dL)	99 (70–298)
Serum creatinine (mg/dL)	0.67 (0.32–7.69)
BTR	5.58 (1.63–11.86)
Serum ammonia (µg/dL)	31 (5–137)
C reactive protein (mg/dL)	0.1 (0–2.4)
VTQ (m/s)	1.34 (0.67–4.32)
FIB-4 index	1.03 (0.12–2.61)
APRI	0.83 (0.09–7.58)
F stage, 0/1/2/3/4/NT	9/114/73/73/178/21

SMI: skeletal muscle index; BMI: body mass index; AST: aspartate aminotransferase; ALT: alanine aminotransferase; BTR: branched-chain amino acid to tyrosine ratio; VTQ: virtual touch quantification; APRI: AST to platelet ratio index; NT: not tested.

**Table 2:** ROC analysis for decreased skeletal muscle index.

	AUROC	Cutoff Value	Sensitivity (%)	Specificity (%)
Age	0.721	62	72.57	62.80
Body mass index	0.805	22.78	85.14	61.77
AST	0.469	57.0	78.87	24.91
ALT	0.532	72.0	89.14	18.77
Serum albumin	0.698	4.0	85.14	54.27
Total bilirubin	0.535	0.80	53.4	54.95
Prothrombin time	0.504	85.7	68.57	38.91
Platelet count	0.579	17.8	76.00	41.64
Total cholesterol	0.521	192	76.57	31.06
Triglyceride	0.576	102	72.57	46.42
Fasting blood glucose	0.466	127	12.0	91.13
Serum creatinine	0.476	0.89	18.29	88.05
BTR	0.563	5.93	68.57	47.78
Serum ammonia	0.478	64	94.51	12.0
C reactive protein	0.548	0	53.71	57.68
VTQ	0.706	1.12	94.86	50.51
FIB-4 index	0.635	0.772	84.57	37.2
APRI	0.564	0.716	64.57	51.54

AUROC: area under the receiver operating curve; AST: aspartate aminotransferase; ALT: alanine aminotransferase; BTR: branched-chain amino acid to tyrosine ratio; VTQ: virtual touch quantification; APRI: AST to platelet ratio index.

**Table 3:** Relationship between VTQ level and baseline data.

	*r_s_*	*p* Value
Age	0.4418	<0.0001
SMI, male	−0.4276	<0.0001
SMI, female	−0.2384	0.0002
Body mass index	−0.0746	0.1071
AST	0.3315	<0.0001
ALT	0.0121	0.7947
Serum albumin	−0.5538	<0.0001
Total bilirubin	0.1994	<0.0001
Prothrombin time	−0.5504	<0.0001
Platelet count	−0.6152	<0.0001
Total cholesterol	−0.3941	<0.0001
Triglyceride	−0.2075	<0.0001
Fasting blood glucose	0.0731	0.1140
Serum creatinine	0.0679	0.1427
BTR	−0.6061	<0.0001
Serum ammonia	0.3191	<0.0001
C reactive protein	0.0559	0.2277
FIB-4 index	0.2122	<0.0001
APRI	0.6011	<0.0001

VTQ: virtual touch quantification; SMI: skeletal muscle index; AST: aspartate aminotransferase; ALT: alanine aminotransferase; BTR: branched-chain amino acid to tyrosine ratio; APRI: AST to platelet ratio index.

**Table 4:** Comparison of baseline characteristics between patients with D-SMI (*n* = 185) and those without D-SMI (*n* = 304).

	Decreased Skeletal Muscle Mass (D-SMI) (*n* = 175)	Non D-SMI (*n* = 293)	*p* Value
Age (years)	67 (25–90)	58 (18–81)	<0.0001
Gender, male/female	76/99	146/147	0.1823
Cause of liver disease	15/113/2/6/39	32/154/2/11/94	0.0988
B/C/B and C/Alcohol/Others			
BMI (kg/m^2^)	20.28 (13.05–31.14)	23.78 (17.21–41.94)	<0.0001
Serum albumin (g/dL)	3.7 (2.6–4.9)	4.1 (2.7–5.1)	<0.0001
Total bilirubin (mg/dL)	0.8 (0.3–5.1)	0.9 (0.2–4.7)	0.2045
Prothrombin time (%)	89.4 (55.5–123)	90.0 (43.3–133.6)	0.8143
Platelet count (×10^4^/mm^3^)	13.7 (3.3–40.8)	15.7 (2.1–50.4)	0.0044
AST (IU/L)	38 (13–125)	35 (12–182)	0.2571
ALT (IU/L)	34 (7–268)	36 (8–247)	0.2449
Total cholesterol (mg/dL)	166 (85–292)	167 (82–448)	0.3264
Triglyceride	85 (27–554)	97 (29–572)	0.1026
Fasting blood glucose	96 (70–298)	101 (73–249)	0.2169
BTR	5.33 (1.63–11.86)	5.83 (1.79–10.46)	0.0219
Serum creatinine (mg/dL)	0.65 (0.32–7.67)	0.68 (0.35–6.5)	0.3808
Serum ammonia	31 (10–105)	30 (5–137)	0.4519
C reactive protein	0 (0–2.3)	0.1 (0–2.4)	0.5844
VTQ	1.64 (0.93–4.32)	1.11 (0.67–4.09)	<0.0001
FIB-4 index	1.15 (0.36–2.61)	0.96 (0.12–2.60)	<0.0001
APRI	0.97 (0.20–4.23)	0.70 (0.09–7.58)	0.0204

SMI: skeletal muscle index; BMI: body mass index; AST: aspartate aminotransferase; ALT: alanine aminotransferase; BTR: branched-chain amino acid to tyrosine ratio; VTQ: virtual touch quantification; APRI: AST to platelet ratio index.

**Table 5:** Multivariate analyses of factors linked to the presence of D-SMI.

Variables	Multivariate Analysis		
	OR	95% CI	*p* Value
BMI (per one kg/m^2^)	1.712	1.523–1.925	<0.0001
Age (per one year)	0.922	0.895–0.953	<0.0001
Platelet count (per one ×10^4^/mm^3^)	1.017	0.969–1.068	0.5003
Serum albumin (per one g/dL)	4.832	2.148–10.872	<0.0001
BTR (per one)	1.374	1.107–1.704	0.0031
VTQ (per one m/s)	0.278	0.167–0.462	<0.0001
FIB-4 index (per one)	0.625	0.384–0.955	0.0425
APRI (per one)	0.455	0.189–1.095	0.0735

OR: Odds ratio; CI: confidence interval; BMI: body mass index; BTR: branched-chain amino acid to tyrosine ratio; VTQ: virtual touch quantification; APRI: AST to platelet ratio index.

## Abbreviations

CLDs: chronic liver diseases; ARFI: acoustic radiation force impulse; VTQ: virtual touch quantification; m/s: meters/second; LC: liver cirrhosis; AST: aspartate aminotransferase; BIA: bio-electronic impedance analysis; APRI: AST to platelet ratio index; NAFLD: non-alcoholic fatty liver disease; NASH: non-alcoholic steatohepatitis; BMI: body mass index; SMI: skeletal muscle mass; D-SMI: decreased skeletal muscle mass; ALT: alanine aminotransferase; ROC: receiver operating characteristic curve; AUROC: area under the ROC; HCV: hepatitis C virus; BTR: branched-chain amino acid to tyrosine ratio; ORs: odds ratios.
